# Developmental prosopagnosia and adaptative compensatory strategies: Case study

**DOI:** 10.1590/S1980-57642009DN20400021

**Published:** 2008

**Authors:** Anair Rodrigues, Sílvia Adriana Prado Bolognani, Sonia Maria Dozzi Brucki, Orlando Francisco Amodeo Bueno

**Affiliations:** 1 Neuropsychologist. Head of Centro Paulista de Neuropsicologia, Departamento de Psicobiologia, UNIFESP-EPM.; 2 Neurologist. Head of Centro Paulista de Neuropsicologia, Departamento de Psicobiologia, UNIFESP-EPM.; 3 Psychologist, Head of Centro Paulista de Neuropsicologia, Departamento de Psicobiologia, UNIFESP-EPM.

**Keywords:** developmental prosopagnosia, prosopagnosia, visual perception, visual agnosia, neuropsychology

## Abstract

Prosopagnosia is a type of visual agnosia with inability to identify faces, usually secondary to brain lesion in associative cortex areas, but there is also a congenital form known as developmental prosopagnosia.

**Objectives:**

To describe a case of developmental prosopagnosia that illustrates the specificity of the pathways for perception of faces in the visual system. Also, we will describe possible mechanisms of recognition used by this patient.

**Methods:**

R.S., a 50 year-old woman, was referred for neuropsychological assessment due to difficulties in perception of familiar faces since childhood, unexplained by any loss of visual acuity.

**Results:**

The exam showed good performance for comprehension, reasoning, concept formation, constructional abilities, criticism, judgment, mental control, memory and visual perception for other kinds of stimuli. No difficulties were seen regarding identification of ethnicity, age and types of animals. The patient was able to match celebrities’ faces in different positions, but could not identify the matching pictures for unknown people.

**Conclusions:**

These findings indicate the patient had developed strategies, throughout life, to recognize familiar faces (relatives, celebrities) from memorized fragments, but still had difficulties in identifying non-familiar faces holistically.

## Abstract (pt)

A prosopagnosia é um tipo de agnosia visual que pode surgir em decorrência de lesão cerebral em áreas do córtex associativo, mas também pode constituir uma condição congênita, também chamada de prosopagnosia de desenvolvimento.

**Objetivos:**

Descrição de um caso de prosopagnosia de desenvolvimento, que ilustra mecanismos específicos de percepção visual, bem como vias alternativas utilizadas no reconhecimento de pessoas.

**Métodos:**

A paciente, 50 anos, foi encaminhada para avaliação neuropsicológica com queixas de dificuldades de percepção visual não justificáveis por perda de acuidade. Desde criança teve dificuldades em reconhecer rostos de pessoas conhecidas.

**Resultados:**

Na avaliação a paciente mostrou bons resultados quanto a capacidade de compreensão e expressão de idéias, raciocínio e conceituação, visuo-construção, crítica, julgamento, controle mental, memória e percepção visual para outras modalidades de estímulos. Também não obteve prejuízos no reconhecimento de etnias, expressões faciais, identificação de faixas etárias e tipos de animais. A paciente foi capaz de parear fotos de rostos familiares famosos em diferentes posições, mas não conseguiu parear rostos não familiares.

**Conclusões:**

O conjunto dos achados indica que a paciente desenvolveu estratégias ao longo da vida para reconhecer rostos conhecidos (familiares, celebridades) a partir de fragmentos memorizados, mas continua tendo dificuldades para identificar holisticamente faces não familiares.

Prosopagnosia is a type of visual agnosia with inability to recognize faces. Studies of the visual system have identified specialized brain pathways for face perception which are not involved in the perception of other classes of stimuli.^1^ Also, the brain has particular ways of recognizing familiar versus non-familiar faces.^2^ Imaging studies have shown that a larger number of brain areas are involved in recognizing familiar faces, such as those areas responsible for memory and emotions.^3^

There are no strict criteria for classification of clinical cases, while prosopagnosia could be related to different perceptual difficulties. It may occur due to injury in the associative areas of the cerebral cortex, such as the fusiform gyrus, and the occipital-temporal areas. Case studies show left hemisphere, right hemisphere or bilateral lesions.^4^ This condition may also be congenital, when it is called developmental prosopagnosia. In this case, the pathology cannot be explained by brain lesion or visual deficiencies such as poor acuity, childhood cataract or myopia.^5,6^ Despite difficulties in identifying faces, patients with prosopagnosia have good IQ scores, general intelligence and are well-adjusted in society.^7^

Our aim in this report was to describe a patient with developmental prosopagnosia, who showed compensatory mechanisms for visual recognition of faces.

## Methods

### Patient

A 50-year-old woman who was a retired teacher with a university degree in Social Sciences was referred for neuropsychological evaluation, complaining of visual perception difficulties since childhood. The patient had difficulties in recognizing the faces of people she knew and reported using other means to identify them: voice tone, body shape, type of hair or gait. She said she could recognize people by their voice or general style, but would have trouble if someone had had a radical change in haircut.

She stated that, during childhood, her mother and sister were her “eyes”, and through them she could build her network of friends. When working as a teacher, she could identify the speaker by their voice (when they were behind her), but when she looked at students' faces she could not tell who the person was. Recently, she went to the beach with her 3 grandsons, and as they were wearing similar dark bathing suits, she could not distinguish them from one another. She had a normal MRI.

### Neuropsychological evaluation

General cognitive functioning was assessed by a comprehensive neuropsychological evaluation using well-known clinical tests. Face perception was measured by an instrument tailored for her assessment using colored photos tasks, as follows:

1. Identification of types and breeds of animals.
2. Discrimination of age and race of unknown people.
3. Identification of emotion and facial expressions of unknown people.
4. Recognition and naming of celebrities.
5. Matching pictures of celebrities in different clothing and poses.
6. Matching pictures of unknown people in different clothing and poses.

Tasks 5 and 6 were the experimental tasks. Each of them contained six pairs of pictures (either unknown people or celebrities) in 2 different outfits and poses. We presented the 12 pictures randomly and asked the patient to match the pictures of the same person.

## Results

Table 1 shows results on global cognitive measures and visual perception tests. Good performance was achieved in tests assessing language, semantic knowledge, comprehension, reasoning, abstract thinking, and visuospatial praxia, as well as memory for verbal (stories) and visual (geometric compositions) stimuli. The patient showed a good ability to perceive objects with incomplete parts, and also to identify drawings of common objects, utensils and animals. She showed moderate impairment on the Hooper Test, which presents picture fragments and assesses the subject’s ability to perform mental rotation and synthesis to identify the whole picture.

**Table 1:** Measures of global cognition and visual perception tests.

Tests	Results
**Wechsler Intelligence Scale - WAIS-R8**	
Comprehension	16 (superior)
Information	13 (above average)
Similarities	12 (normal)
Vocabulary	15 (superior)
Picture arrangement	10 (normal)
Block design	11 (normal)
W**echsler Memory Scale - WMS-R9**	
Logical memory immediate	21 (normal)
Logical memory delayed	20 (normal)
Visual reproduction immediate	34 (normal)
Visual reproduction delayed	24 (normal)
**Visual perception tests**	
Figure completion (WAIS-R)	10 (normal)
Boston Naming Test^10^(figure identification)	60 (normal)
Hooper Visual Organization Test^10^	20 (low average)

Table 2 shows ‘pass’ or ‘fail’ scores on face recognition tasks. The patient was able to identify race, ages and emotions in pictures of unfamiliar people, as well as recognize faces of Brazilian actresses, actors and politicians. She matched all celebrities’ faces from several scrambled pictures successfully, except for one person, whose pictures had very different angles: one was a front close up smiling and the other was a side shot with a more neutral face. She was not successful in matching pictures with faces of unknown people.

**Table 2:** Results on visual recognition and face recognition tasks.

	item 1	item 2	item 3	item 4	item 5	item 6
Animals	+	+	+	+	+	+
Ages and races	+	+	+	+	+	+
Emotions and expressions	+	+	+	+	+	+
Brazilian celebrities	+	+	+	+	+	+
Celebrities matching	+	-	+	+	+	+
Unknown faces matching	-	-	-	-	-	-

Scores are represented as pass (+) or fail (-).

During the assessment sessions, the patient made several comments about her performance in informal activities of face recognition that are commonly reported by prosopagnosic patients in the literature:

- She used distinctive facial features to recognize people.
- She thought one of the pairs “seemed” to be the same person, but as one was smiling and the other was not, she decided they were different people.
- She was able to recognize pictures of actors in papers and magazines. However, she was unable to identify them properly when they appeared in a movie, or to follow the characters’ story line.

## Discussion

Case studies of patients with prosopagnosia have tended to show strong evidence that the visual system has different pathways for recognizing faces or objects. Also well accepted is the assumption that prosopagnosia can be a result of deficits in different mechanisms underlying face perception, where these are not common to all individuals studied.^11^ Such variations can be attributed to different methods of assessment, developmental issues, and also individual cognitive experiences in life (cultural, work activities and compensatory strategies developed).

In this study we used informal tests and showed that, for this patient, face-recognition impairments were more evident when the patient had to match two different pictures of an unknown person. She was unable to perform any item of this task, which presented the same face in different positions and context: for example, a view that was slightly from the side had to be matched with a front view, or a smiling face with a non-smiling shot. However, the patient had much less difficulty in performing the same task with familiar faces (celebrities), failing to recognize only one pair of faces as being the same person. These results were different to the prosopagnosic patients reported by Dobel et al. whose patients were unable to recognize famous faces. Akin to our patient, judgment of emotional expression was impaired to a lesser degree.^7^

These findings suggest that, because of multiple previous experiences, facial traits of known people had already being stored as identifiable faces, thus allowing the recognition of celebrities. However, although this patient was able to recognize partial features, her impairment in matching non-familiar faces may be due to an inability to see a face as a non-decomposable whole, corroborating the idea that holistic processing of features is one of the particularities of facial perception mechanisms.^1,6^

This patient’s performance on the tasks suggests that, throughout life, she had developed internal compensatory strategies to recognize familiar faces by memorizing fragments of facial features, although she was unable to perform holistic identification of non-familiar faces.^6,7,11^ Also, this case shows that developmental prosopagnosia may exist in the absence of other cognitive deficits and without difficulties in recognizing objects.
